# New Method of Administering Quinine

**Published:** 1854-04

**Authors:** 


MONTHLY MEMORANDA.

*New method of administering Quinine.*—The addition of Tartaric acid to Quinine renders it more soluble and favors its absorption. It is supposed that a solution of equal parts of the salt and acid is equal in power to a similar solution, where the salt alone is used. For instance, if ten grains of each are dissolved, the solution would be equal in strength to a scruple of quinine, the dose being the same. Dr. Bertella has written an able article on the subject, and, in addition to the facts above stated, he maintains that the salt is not decomposed as in some other forms of solution. Beside other advantages it would prove more economical; quite a desideratum in malarious districts.

